# Association of *Mycoplasma hominis* infection with prostate cancer

**DOI:** 10.18632/oncotarget.256

**Published:** 2011-04-04

**Authors:** Yulia A. Barykova, Denis Yu. Logunov, Maxim M. Shmarov, Andrei Z. Vinarov, Dmitry N. Fiev, Natalia A. Vinarova, Irina V. Rakovskaya, Patricia Stanhope Baker, Inna Shyshynova, Andrew J. Stephenson, Eric A. Klein, Boris S. Naroditsky, Alexander L. Gintsburg, Andrei V. Gudkov

**Affiliations:** ^1^ N.F. Gamaleya Research Institute for Epidemiology and Microbiology, Moscow, Russia; ^2^ I.M. Sechenov First Moscow State Medical University, Moscow, Russia; ^3^ Cleveland BioLabs, Inc., Buffalo, NY, USA; ^4^ Glickman Urological and Kidney Institute, Taussig Cancer Center, Cleveland Clinic, Cleveland, OH, USA; ^5^ Roswell Park Cancer Institute, Buffalo, NY, USA

**Keywords:** PCR, diagnostics, prostate biopsies, prostate intraepithelial neoplasia, benign prostate hyperplasia

## Abstract

The origin of chronic inflammation preceding the development of prostate cancer (PCa) remains unknown. We investigated possible involvement of mycoplasma infection in PCa by screening prostate biopsies from two groups of Russian men undergoing PCa diagnosis. *M. hominis* was detected by standard PCR in 15% of the 125 patients in the first group and by quantitative real-time PCR in 37.4% of the 123 men in the second group. In both groups, stratification of patients according to diagnosis showed that *M. hominis* was present at three times higher frequency in patients with PCa than in those with benign prostatic hyperplasia. No *M. hominis* was detected in the prostates of 27 men without detectable prostate disease. In addition, PCa-positive men had higher titers of antibodies against *M. hominis* and average PSA levels were higher in *M. hominis*-positive men. These data, together with previous observations linking mycoplasma infection with cell transformation, genomic instability and resistance to apoptosis, suggest that *M. hominis* infection may be involved in PCa development and may, therefore, be a potential PCa marker and/or target for improved prevention and treatment of this disease.

## INTRODUCTION

Prostate cancer (PCa) is the most common cancer and the second leading cause of cancer-related death in men of the Western world [1]. For a number of cancers, including PCa, chronic inflammation associated with infections has been defined as an important cancer-promoting condition [2-5]. Identification of additional chronic infections associated with cancer, as well as the mechanisms underlying their cancer-promoting activity, will be important for developing new approaches for cancer prevention, diagnosis and treatment [3].

Despite indications that chronic infections are important etiological factors for PCa [2, 4, 5], only one such agent has been reported to date. Infection with the xenotropic retrovirus XMRV was shown to be associated with PCa, although only in a minor proportion of PCa patients [6]. Therefore, the major factors responsible for PCa-promoting chronic inflammation have yet to be defined. In the current study, we tested the possible involvement of mycoplasmas. Mycoplasmas are parasitic bacteria that infect vertebrates. Mycoplasma infection affects the cellular metabolism and physiology of the host organism [7] and is associated with diseases in humans and animals [7, 8]. Nevertheless, these microorganisms are generally regarded as normal commensals of the human urogenital microflora which only become pathogenic under specific, relatively rare conditions [7, 8]. This is supported by the high frequency of chronic, asymptomatic infection of humans by these organisms [7].

The chronic nature of mycoplasma infection suggests that it could generate chronic inflammation with pro-cancerous effects. Numerous studies have shown that chronic mycoplasma infection of cell cultures can result in genetic instability and malignant transformation [9-14]. Mycoplasma infection was shown to increase the *in vitro* invasiveness and *in vivo* metastasis of different human tumor cells [10, 12, 14]. Mycoplasma infection was also shown to act as an oncogene capable of cooperating with H-*ras* or c-*myc* to induce transformation of embryonic cells [15]. Moreover, mycoplasma infection both suppresses p53 activity and activates NF-κB [16], thereby mimicking two of the most common features of tumor cells [17, 18].

Taken together, these properties of mycoplasmas led us to hypothesize that mycoplasma infection of prostate tissue may be associated with PCa and play a role in promoting development of PCa through generation of chronic inflammation. To test this hypothesis, we screened prostate tissue samples from men suspected of PCa due to elevated PSA level for presence of mycoplasma DNA. Comparison of these results with those from control “normal” prostates and stratification of the data in terms of patient diagnosis revealed a statistically significant correlation between *M. hominis* infection and PCa (*p*<0.0001). These results suggest that this infectious agent is involved in PCa development and may, therefore, be a potential target for improved prevention, detection and/or treatment of PCa.

## RESULTS AND DISCUSSION

### Detection of mycoplasma infection in prostate tissue from patients suspected of PCa

**Figure 1:**
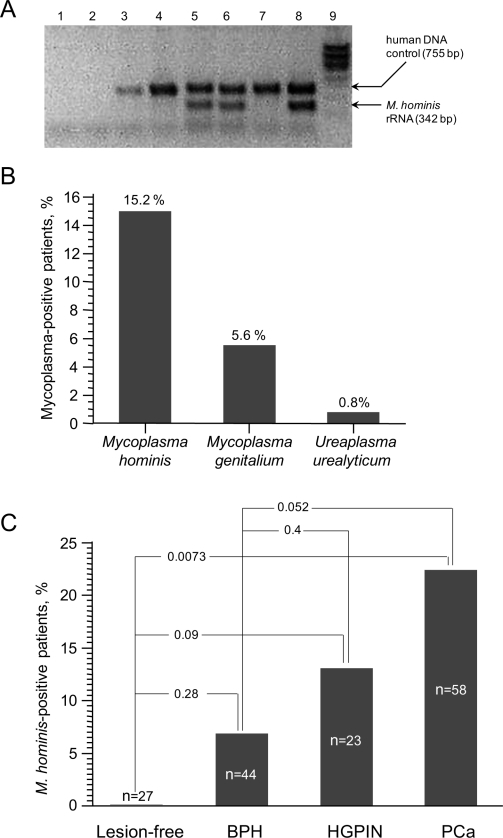
Detection of mycoplasma DNA in prostate tissue from Russian Patient Set 1 using standard PCR **A.** Results of standard PCR assay for detection of an *M. hominis* DNA sequence (16S rRNA gene) and a control human DNA sequence (β-actin gene). 1 – no DNA template; 2 – “mock” DNA extraction; 3-7 – DNA from prostate biopsies of patients suspected of PCa; 8 – positive control (human DNA mixed with *M. hominis* DNA); 9 – DNA ladder. **B.** Frequency of detection of three *Mycoplasma* species in prostate biopsies (n=250, 2 from each of 125 patients) using standard PCR assays. The percentage of patients for which the PCR assay was positive in at least one biopsy sample is indicated. **C.** Frequency of detection of *M. hominis* DNA by standard PCR in prostate biopsies from patients in the indicated diagnosis categories and in prostate samples from negative control men (“lesion-free”).

To determine whether mycoplasma infection is associated with PCa, we screened prostate samples by PCR for the presence of DNA sequences of the mycoplasma species most frequently found in the human urogenital tract: *M. hominis, M. genitalium* and *U. urealitycum*. We tested 250 biopsy samples, one each from the left and right lobes of the prostates of 125 Russian patients suspected of PCa due to elevated PSA levels (Patient Set 1). This group included diagnoses ranging from benign prostatic hyperplasia (BPH) to high-grade prostatic intraepithelial neoplasia (HGPIN) to PCa, as determined by histopathological evaluation of additional biopsy samples. We also evaluated 162 negative control samples of prostate tissue taken from men that had normal prostate histology and PSA levels and died from non-cancer-related causes (6 biopsy-sized samples from each of 27 men). Each PCR assay was designed to simultaneously detect a DNA sequence specific to the mycoplasma species of interest and a control human DNA sequence. Figure 1A shows representative results for detection of *M. hominis* sequences in genomic DNA prepared from human prostate tissue. In testing the 250 patient biopsy samples, we found that 21.6% of the 125 patients with suspected PCa were positive for one of the tested species of mycoplasma in at least one biopsy sample (Figure 1B). *M. hominis* infection was most prevalent (found in 15.2% of the patients), while *M. genitalium* and *U. urealitycum* were only detected in 5.6% and 0.8% of the patients, respectively. Notably, none of the three mycoplasma species were detected in any of the 162 negative control prostate samples or in rectal smears taken from 105 of the 125 patients suspected of PCa to control for possible contamination of prostate tissue samples with rectal flora.

The presence of *M. hominis* and *U. urealyticum* in prostate tissue was confirmed by seeding tissue on selective nutrient culture media. *M. genitalium* was not analyzed using this method since its cultivation is reportedly extremely difficult [19]. In testing the same 250 biopsies that were analyzed by PCR, we found that the frequency of identification of *U. urealitycum* was the same with the cultivation assay as with PCR (0.8%). *M. hominis* was also detected by cultivation, although at a lower frequency than by PCR (6.3% vs. 15.2%). This difference could be due to the exacting requirements for cultivation of *M. hominis* or detection of DNA from dead bacteria in the PCR assay. Despite the lower sensitivity of the cultivation assay, growth of mycoplasma from at least some patient samples confirms the presence of active mycoplasma infection in prostate tissue. Notably, none of the 162 negative control prostate samples were positive in the *M. hominis* or *U. urealyticum* cultivation assays.

Since *M. hominis* was detected much more frequently than the other tested mycoplasma species, we focused specifically on this species for the remainder of the study. As shown in Figure 1C, when the 125 patients in Patient Set 1 were divided based on their histopathologically determined diagnosis, it was revealed that the frequency of *M. hominis* infection increased with the severity of the diagnosis (6.8%, 13.0% and 22.4% in BPH, HGPIN and PCa patients, respectively, as compared to 0% in the 27 control patients with normal “lesion-free” prostates). HGPIN is increasingly considered a precursor to PCa in many, if not most, cases. In contrast, there is no clear association of BPH with PCa. Our data shows that *M. hominis* was detected 3-times more frequently in patients with either HGPIN or PCa (20%) than in patients with BPH (6.8%). Looking at the data in terms of the occurrence of HGPIN and PCa in *M. hominis*-positive versus *M. hominis*-negative groups, it is clear that *M. hominis* infection is associated with development of PCa. 84.2% of subjects found to be *M. hominis*-positive were diagnosed with either HGPIN or PCa, while only 57.6% of *M. hominis*-negative PSA-positive subjects fell into these diagnosis categories (*p*=0.033).

### Analysis of *M. hominis* infection in prostate tissue using quantitative real-time PCR

**Figure 2:**
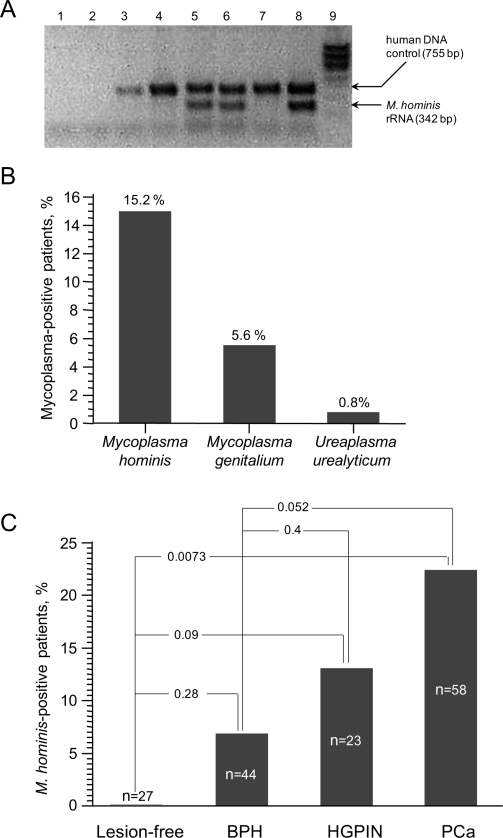
Association of *M. hominis* infection with HGPIN and PCa **A.** Frequency of detection of *M. hominis* DNA by qPCR in prostate biopsies from patients of Russian Patient Set 2 in the indicated diagnosis categories and in prostate samples from negative control men (“lesion-free”). **B.** Quantitation of *M. hominis* 16S rRNA gene copies by qPCR in *M. hominis*-positive prostate biopsies from patients in Russian Patient Set 2 with BPH (n=15 biopsies from 12 patients), HGPIN (n=28 biopsies from 17 patients) or PCa (n=25 biopsies from 17 patients). Each point indicates the concentration of *M. hominis* 16S rRNA DNA in a particular biopsy sample and the horizontal lines indicate the average value for each diagnosis group.

To confirm the findings described above, we used a quantitative real-time PCR (qPCR) assay to detect *M. hominis* 16S rRNA gene sequences in an independent set of patient samples (Patient Set 2). We analyzed 246 new prostate biopsy samples (one each from the right and left prostate lobes of 123 Russian patients suspected of PCa due to elevated PSA level) as well as the same 162 “normal” negative control prostate tissue samples that we used in our initial standard PCR screen. We detected *M. hominis* DNA in at least one biopsy sample from 46 (37.4%) of the 123 patients suspected of PCa, but not in any of the 27 negative control patients. When Patient Set 2 was divided according to diagnosis, the qPCR data indicated that 53% of patients with HGPIN and 54.8% of patients with PCa were infected with *M. hominis* (Figure 2A). In contrast, none of the negative control patients and only 20% of patients with BPH were *M. hominis*-positive. The association of *M. hominis* infection with HGPIN and PCa was highly statistically significant when compared to either normal “lesion-free” controls (*p*=0.0001) or BPH patients (*p*=0.002). Analysis of the qPCR data in terms of the occurrence of HGPIN and PCa in *M. hominis*-positive versus –negative groups showed that 74% of *M. hominis*-positive patients had HGPIN or PCa, while only 38% of *M. hominis*-negative patients were in these diagnosis groups (*p*<0.002). Thus, *M. hominis* infection was associated with a 2-fold higher risk of PCa in this patient population.

The greater frequency of *M. hominis* detection by qPCR in Russian Patient Set 2 as compared to standard PCR in Russian Patient Set 1 (for example, 54.8% versus 22.4% for PCa samples) is likely due to the greater sensitivity of the qPCR technology rather than differences in the presence of mycoplasma in the two patient populations. This is supported by our finding of a ~3:1 ratio of *M. hominis* presence in HGPIN+PCa patients versus BPH patients in both the standard and qPCR studies.

In addition to finding that the rate of *M. hominis* infection is greater in PCa and HGPIN patients than in BPH or “normal” patients, we identified a similar correlation for the degree of *M. hominis* infection. Using our qPCR assay we found that the mean concentration of *M. hominis* DNA in samples from BPH patients was less than 4x10^4^ copies/ml (Figure 2B). In comparison, the mean concentration of *M. hominis* DNA in HGPIN samples was more than 10-fold higher (5.5x10^5^ copies/ml) and PCa samples showed an even higher level of infection (9.1x10^5^ copies/ml). This positive correlation between the quantity of *M. hominis* in the prostate and diagnoses of HGPIN or PCa provides additional support for the association between *M. hominis* and PCa and is consistent with the possibility that *M. hominis* infection plays a role in development of PCa.

### Detection of anti-*M. hominis* antibodies in the serum of patients suspected of PCa

**Figure 3:**
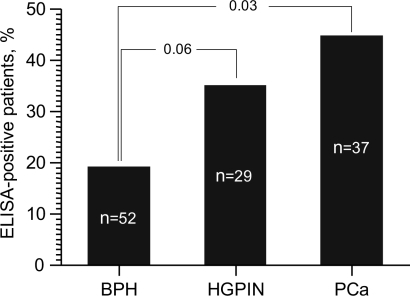
ELISA-based detection of anti-*M. hominis* IgG in the serum of patients suspected of PCa ELISA was performed for 118 patients out of the 123 patients in Russian Patient Set 2 for which serum was available. The percentage of patients in BPH (n=52), HGPIN (n=29) and PCa (n=37) diagnosis categories that were positive by ELISA is shown.

As an independent means to confirm mycoplasma infection of patient prostate tissue, we tested serum from 118 men by ELISA for the presence of IgG antibodies against recombinant *M. hominis* protein p120. The negative control patients were not analyzed by ELISA since their serum was not available. As shown in Figure 3, 35% and 44.8% of PCa and HGPIN patients were positive for anti-*M. hominis* IgG, respectively. In contrast, only 19% of patients with BPH were positive. The correlation of positive *M. hominis*-specific ELISA results with HGPIN/PCa as compared to BPH was statistically significant (*p*=0.042) For 75% of the patients analyzed by both ELISA and real-time PCR, the two assays gave consistent results (*M. hominis*-positive or -negative). The two assays gave disparate results in the other 25% of cases, perhaps due to the different biological materials analyzed and/or the different natures and sensitivities of the methods. Thus, while the ELISA assay provided confirmation of the association between *M. hominis* infection and diagnoses of HGPIN and PCa, qPCR was found to be the most sensitive method of *M. hominis* detection. Nevertheless, if the association between *M. hominis* and PCa is borne out, the humoral immune response to *M. hominis* could provide a clinically useful diagnostic marker reflective of disease development and stage.

### Preferential localization of Mycoplasma infection in the left lobe of the prostate

**Figure 4:**
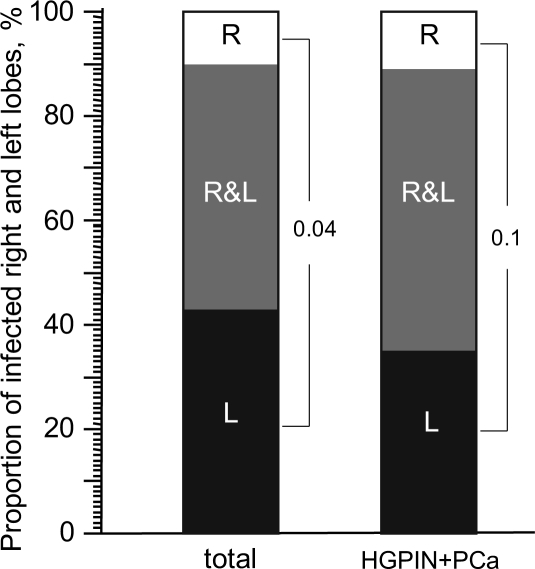
The proportion of *M. hominis*-positive patients in which both prostate lobes were positive (R&L) or only the left (L) or right (R) lobe was positive Data is from qPCR analysis of Russian Patient Set 2 and is shown for *M. hominis*-positive patients of all diagnosis groups (“total”, left column, n=46) or the HGPIN+PCa groups only (right column, n=34).

Since we had biopsy samples from each of the two lobes of the prostate from patients suspected of PCa, we used our data from Patient Set 2 to determine whether mycoplasma infection showed any bias in localization. As illustrated in Figure 4, for those patients in which *M. hominis* was detected, it was found in both lobes of the prostate in 46% of cases, only in the right lobe in 11% of cases, and only in the left lobe in 43% of cases. Thus, when only one lobe of the prostate was infected, the infection occurred in the left lobe 4 times more often than in the right lobe (*p*=0.04). A similar pattern was observed when only patients diagnosed with HGPIN or PCa were analyzed, although this did not reach statistical significance. In this case, infection of both lobes was found in 53% of *M. hominis*-positive patients, while infection of only the right or left lobe was seen in 12% and 35% of patients, respectively. The observed bias in infection localization might be due to the anatomy of the prostate, particularly in terms of blood flow through the tissue. This possibility is supported by a study of 300 patients with chronic prostatitis in which inflammatory changes were detected in both prostate lobes in 42% of cases, only in the right lobe in 13.3% of cases, and only in the left lobe in 44.7% of cases [20, 21]. In this study, it was postulated that anatomic features resulting in venous stagnation of blood contributed to prostate tissue damage and its preference for the left lobe. In addition, clinicians have noted that inflammatory changes are more expressed and deep in the left lobe of the prostate than the right, even if both lobes are affected [20, 21]. Such tissue changes appear to promote penetration of pathogenic organisms into the prostate tissue. For example, in patients with chlamydia prostatitis, pathological changes are both more common and more severe in the left prostate lobe than the right [20].

### Relationship between PSA level and M. hominis infection

**Figure 5:**
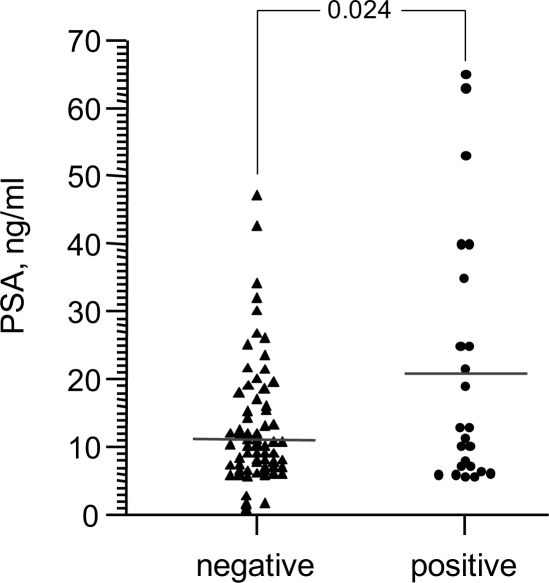
Serum PSA levels in patients suspected of PCa (Russian Patient Set 1) that were found to be *M. hominis*-negative (n=99) or –positive (n=26) by standard PCR Each point represents an individual patient and the horizontal line indicates the mean within each group.

Prostate specific antigen (PSA) is a protein produced by the prostate gland that is normally present in the blood at very low levels. Elevation of the serum PSA level is associated with PCa and other prostate disorders [22]. Measurement of serum PSA level is widely used as a screening tool for early detection of PCa, although it frequently provides false positive results [22]. To further explore the association between *M. hominis* infection and PCa, we investigated whether a correlation existed between presence of *M. hominis* and serum PSA level. Serum PSA levels were determined for the 125 patients in Patient Set 1 at the time that biopsies were taken. Separation of these patients into *M. hominis*-positive and -negative groups revealed a positive correlation between PSA level and *M. hominis* infection (Figure 5). The average PSA level in *M. hominis*-positive patients was 21 ng/ml, while the average level in *M. hominis*-negative patients was 12 ng/ml. This difference was statistically significant (*p*=0.024) and provides further support for association of *M. hominis* infection with development of PCa.

### Detection of *M. hominis* in the prostate tissue of American men suspected of PCa

To extend our findings beyond Russian patient populations, we performed a small pilot study to investigate the presence of *M. hominis* in American men suspected of PCa. Prostate biopsies (n=116, 2 from each of 58 Cleveland Clinic patients) were screened using the *M. hominis* 16S rRNA qPCR assay. While the number of analyzed biopsies was too small to make statistically reliable conclusions regarding the correlation between mycoplasma infection and diagnosis, we did find that a substantial proportion of the samples (15.5%) were *M. hominis*-positive (Table 1). These preliminary data demonstrate that infection of the prostate by this species of mycoplasma is not an endemic characteristic of Russian men and, therefore, may have general importance in consideration of potential strategies for prevention and treatment of PCa.

**Table 1:** Occurrence of *M. hominis*-positive samples among prostate biopsies of American patients

Histopathology evaluation	Number of patients	*M.hominis* positive (number)	*M.hominis* positive (%)
PCa positive	27	5	18.5
PCa negative	31	4	12.9
Total	58	9	15.5

## CONCLUSIONS

Despite considerable well-justified speculation that mycoplasma infection might play a role in cancer promotion, the current study provides, to our best knowledge, the first demonstration of a statistically significant association between mycoplasma infection and cancer development. Our results are notable in that *M. hominis* infection was detected by qPCR in a substantial fraction of men diagnosed with HGPIN or PCa (>50%), yet only 20% of men with BPH and 0% of men with apparently normal prostates. These differences were highly statistically significant, but will need to be confirmed in additional large-scale epidemiological studies involving men of different nationalities. If it is borne out that *M. hominis* infection is a predisposing factor in PCa, *M. hominis* will become a promising new target for PCa diagnosis, prevention and treatment. In particular, if *M. hominis* is found to play a causative role in PCa, early detection and eradication of *M. hominis* infection could become a routine PCa-preventive approach. However, even if *M. hominis* infection is only “passively” associated with PCa, it could be used as an additional diagnostic marker to improve the accuracy of PSA-based diagnoses.

## MATERIALS AND METHODS

### Prostate tissue samples

For *Russian Patient Sets 1 and 2*, prostate tissue samples were obtained in the urological clinic of the Setcheinov Moscow Medical Academy from 125 (Set 1) and 123 (Set 2) patients suspected of PCa due to elevated PSA level. Patient age ranged from 45 to 83 years (mean = 65.5±1.02 years). Transrectal polyfocal prostate biopsies were performed under ultrasonic pointing using sterile biopsy needles. For each patient, twelve main columns of prostate tissue were taken for morphological and histological studies and two additional samples (one each from the peripheral left and right prostate lobes) were taken for our research. Thus, 496 acerate biopsy samples of prostate tissue were obtained. In addition, rectal smears were taken from 105 of the patients in Set 1 for use as controls. *Negative controls* (n=27) were Russian men (age 33-60, mean = 55.2±2.5 years) that had normal PSA levels and prostate histology and died from causes unrelated to cancer. For each control prostate, 6 tissue samples of biopsy size were taken from the same zones biopsied in patients (162 total samples). For both patients and controls, to reduce the possibility of degradation of mycoplasmal DNA within the tissue, DNA was prepared from prostate tissue either immediately upon collection or after no more than 1-2 days of storage at −70 degrees. *American Patient Set*: Prostate tissue samples were obtained at the Urological Institute of the Cleveland Clinic from 58 patients suspected of PCa due to elevated PSA level. Transrectal polyfocal prostate biopsies were performed under ultrasonic pointing. Two tissue samples were taken from each patient (one each from the peripheral left and right prostate lobes). Histopathological studies leading to diagnosis and all PCR and qPCR studies were performed in a double-blinded manner.

### DNA extraction

DNA was prepared from tissue samples using Proteinase K treatment (1 μg/ml final concentration of Proteinase K, incubated at 65°C for 1.5-2 hours) followed by DNA extraction using the DNA-sorb-B reagent set (InterLabService, Moscow, Russia) according to the manufacturer's instructions. The obtained purified DNA was stored at −20°C.

### Standard PCR assays for detection of mycoplasma DNA in Patient Set 1 samples

PCR assays were designed to simultaneously detect specific DNA sequences of either *M. hominis, M. genitalium*, or *U. urealyticum* along with a human DNA sequence (as an internal control for DNA quality and quantity). Primer sequences are available upon request. Plasmid DNAs containing *M. hominis, M. genitalium* or *U. Urealitycum* sequences were used as positive controls. “Hot start” PCR was performed using TC-Taq-DNA-polymerase. Amplification conditions were: 1 cycle at 95°C – 10 min; 45 cycles at 94°C – 10 sec, 61-63°C (depending on mycoplasma species) –10 sec, 72°C – 10 sec. Reaction products were electrophoresed in 1.5% agarose gels containing 1 μg/ml ethidium bromide.

### Quantitative real-time PCR (qPCR) assay for detection of *M. hominis* 16S rRNA sequences in Patient Set 2 samples

We designed a qPCR assay for quantitative detection of *M. hominis* 16S rRNA sequences. The specific primers and “TaqMan” probe for the *M. hominis* 16S rRNA qPCR assay were designed in accordance with the rules provided by Primer Express (Applied Biosystems): forward primer: 5'-aaa-aga-tga-ggg-tgc-gga-aca-3'; reverse primer: 5'-ttc-cct-act-gct-gcc-tcc-cgt–3'; probe: 5' - FAM- tgg-ccg-ttc-agt-ctc-tcg-acc-cgg-cta BTQ1– 3'. The probe was marked on the 5'-end with the fluorescent reporter FAM and on the 3'-end with the quencher BTQ1. Amplification conditions were: 1 cycle at 95°C – 300 sec; 50 cycles at 60°C – 50 sec, 95°C – 20 sec. Analysis was carried out using the ANK-32 tool (IAnP RAS Institute for Analytical Instrumentation Russian Academy of Science and Bauman Moscow State Technical University, Russia).

### Mycoplasma cultivation assays

Presence of *M. hominis* and *U. urealyticum* in prostate tissue samples was determined by seeding tissue on selective nutrient culture media enriched with arginine or urea, respectively. Media also contained a colorimetric indicator of mycoplasma growth. Seeding was performed using standard microbiological techniques as described in [19] with cultivation under anaerobic conditions at 37°C with an abundance of CO^2^.

### Detection of anti-*M. hominis* IgG in blood serum from patients

An enzyme-linked immunosorbent assay (ELISA) was used to detect IgG antibodies specific for *M. hominis* in patient sera (“Vector Best,” Russia), according to the manufacturer's instructions.

### Statistics

Reliability of the correlations among the groups under study was determined using χ^2^ criterion (for 2-by-2 tables with Fisher's exact test). Credibility towards the mean age difference was determined using Student's criterion.
